# Performance of several simple, noninvasive models for assessing significant liver fibrosis in patients with chronic hepatitis B

**DOI:** 10.3325/cmj.2015.56.272

**Published:** 2015-06

**Authors:** Xianghua Zeng, Cheng Xu, Dengming He, Maoshi Li, Huiyan Zhang, Quanxin Wu, Dedong Xiang, Yuming Wang

**Affiliations:** 1 Department for Infectious Diseases, Southwest Hospital, Third Military Medical University, Chongqing, People's Republic of China; 2 Chongqing Key Laboratory of Research of Infectious Diseases, Chongqing, People's Republic of China; 3 Liver Disease Diagnosis and Treatment Center, 88th Hospital of Chinese People’s Liberation Army, Tai’an, People's Republic of China; 4 77608 Troops of Chinese People’s Liberation Army, Lhasa, Tibet

## Abstract

**Aim:**

To compare the performance of several simple, noninvasive models comprising various serum markers in diagnosing significant liver fibrosis in the same sample of patients with chronic hepatitis B (CHB) with the same judgment standard.

**Methods:**

A total of 308 patients with CHB who had undergone liver biopsy, laboratory tests, and liver stiffness measurement (LSM) at the Southwest Hospital, Chongqing, China between March 2010 and April 2014 were retrospectively studied. Receiver operating characteristic (ROC) curves and area under ROC curves (AUROCs) were used to analyze the results of the models, which incorporated age-platelet (PLT) index (API model), aspartate transaminase (AST) to alanine aminotransferase (ALT) ratio (AAR model), AST to PLT ratio index (APRI model), γ-glutamyl transpeptidase (GGT) to PLT ratio index (GPRI model), GGT-PLT-albumin index (S index model), age-AST-PLT-ALT index (FIB-4 model), and age-AST-PLT-ALT-international normalized ratio index (Fibro-Q model).

**Results:**

The AUROCs of the S index, GPRI, FIB-4, APRI, API, Fibro-Q, AAR, and LSM for predicting significant liver fibrosis were 0.726 (*P* < 0.001), 0.726 (*P* < 0.001), 0.621 (*P* = 0.001), 0.619 (*P* = 0.001), 0.580 (*P* = 0.033), 0.569 (*P* = 0.066), 0.495 (*P* = 0.886), and 0.757 (*P* < 0.001), respectively. The S index and GPRI had the highest correlation with histopathological scores (r = 0.373, *P* < 0.001; r = 0.372, *P* < 0.001, respectively) and LSM values (r = 0.516, *P* < 0.001; r = 0.513, *P* < 0.001, respectively). When LSM was combined with S index and GPRI, the AUROCs were 0.753 (*P* < 0.001) and 0.746 (*P* < 0.001), respectively.

**Conclusion:**

S index and GPRI had the best diagnostic performance for significant liver fibrosis and were robust predictors of significant liver fibrosis in patients with CHB for whom transient elastography was unavailable.

Liver fibrosis is a common pathological process in various chronic liver diseases, including chronic hepatitis B (CHB). In patients with CHB, early detection of liver fibrosis is crucial for therapy planning and prognosis estimation. In particular, the presence of significant liver fibrosis is a strong indication for initiating antiviral therapy (1,2). However, liver biopsies, the gold standard for staging fibrosis, are not performed in all hospitals (especially in primary care) because of their invasiveness, sampling errors, and complications. In addition, biopsies are not appropriate for monitoring disease progression. Transient elastography (FibroScan; Echosens, Paris, France), which measures liver stiffness, is increasingly being recognized as an excellent tool for assessing the degree of fibrosis (3,4). FibroScan’s noninvasive nature, reproducibility, and diagnostic performance have also made it increasingly popular. However, not all hospitals have the means to purchase such expensive equipment.

Accordingly, in recent years combinations of serum biomarkers of liver fibrosis have been a hot research topic. Several serological models for liver fibrosis (5-7) that incorporate direct or indirect biomarkers have been developed as alternatives to biopsy. These models reportedly vary considerably in their ability to diagnose fibrosis, and their results are conflicting (8,9). The present study assessed the effectiveness of the following seven fibrosis models, all of which comprise routine serum biomarkers and were found to have predictive value for significant liver fibrosis: age-platelet (PLT) index (API) (10), aspartate transaminase (AST) to alanine aminotransferase (ALT) ratio (AAR) (10,11), AST to PLT ratio index (APRI) (10,11), γ-glutamyl transpeptidase (GGT) to PLT ratio index (GPRI) (11), GGT-PLT-albumin (ALB) index (S index) (12), age-AST-PLT-ALT index (FIB-4) (13), and age-AST-PLT-ALT-international normalized ratio (INR) index (Fibro-Q) (14).

## Methods

### Baseline patients' characteristics

This retrospective study included 308 consecutive patients with CHB attending the Department of Infectious Diseases, Southwest Hospital, Chongqing, China between March 2010 and April 2014. The criterion for diagnosis of CHB was serum hepatitis B surface antigen positive for more than 6 months (2). All enrolled patients underwent liver biopsy, FibroScan, and laboratory tests within 2 days of one another. Because liver stiffness measurement (LSM) values can be influenced by inflammation (15,16), patients with serum ALT concentrations more than five times higher than the upper limit of normal (42 IU/L in both sexes) were excluded. We also excluded patients with concurrent infection with other viruses, decompensated cirrhosis, hepatocellular carcinoma, hepatic failure, and other liver diseases. The research was conducted in accordance with the ethical guidelines of the Declaration of Helsinki 2008 and was approved by the ethics committee of our hospital. Each patient gave their written informed consent.

### Liver biopsy

Ultrasonography-guided percutaneous liver biopsy was performed using a 16 G disposable needle (Hepafix, B. Braun, Melsungen, Germany) under local anesthesia. Specimens of minimum length 10 mm were immediately fixed in 10% formalin for further analysis (17,18). All biopsy samples were reviewed independently by two histopathologists who were blinded to the clinical data. If they failed to reach an agreement, a third histopathologist reviewed the material.

Liver fibrosis was classified into five stages according to the METAVIR scoring system as follows (19): F0, no fibrosis; F1, mild fibrosis without fibrous septum; F2, fibrosis with a few fibrous septa; F3, numerous septa without cirrhosis; and F4, cirrhosis. Significant liver fibrosis was defined as F2 or greater (F≥2) (1).

### FibroScan

LSM values were obtained with FibroScan by an experienced operator (more than 40 000 measurements). The examinations were performed in accordance with the user manuals and steps described previously (15,20). LSM values are expressed as kilopascals (kPa). When the success rate (number of valid acquisitions divided by number of attempts) was over 60% and the ratio of interquartile range to median under 30%, a median value of 10 successful measurements was considered valid.

### Laboratory procedures

Fasting blood serum samples were used for laboratory tests. Serologies for hepatitis B surface antigen were detected with an automated blood analyzer (Advia-Bayer, Leverkusen, Germany). Routine blood (measured by XT-2000i, Sysmex, Kobe, Japan) and biochemistry variables (measured by 7600 Series, Hitachi, Tokyo, Japan) were assessed, including PLT count, serum ALT, AST, GGT, ALB, total bilirubin (TBIL), alkaline phosphatase (ALP), and globulin concentrations. Using the formulas shown in Table 1, the API (10), AAR (10,11), APRI (10,11), GPRI (11), S index (12), FIB-4 (13), and Fibro-Q (14) were calculated, all of these being considered noninvasive models for evaluating the degree of liver fibrosis.

**Table 1:** Formulas for noninvasive fibrosis models using routine laboratory tests*

Fibrosis models	Formulas
AAR	AST (IU/L)/ALT (IU/L)
GPRI	GGT (IU/L)/PLT (IU/L)
S index	1000 × GGT (IU/L)/(PLT (10^9^/L) × ALB^2^ (g/L))
APRI	(AST (IU/L)/ULN (IU/L)/PLT (10^9^/L)) × 100
FIB-4	(age (years) × AST (IU/L))/(PLT (10^9^/L) × ALT^1/2^ (IU/L))
Fibro-Q	(10 × age (years) × AST (IU/L) × INR)/(PLT (10^9^/L) × ALT (IU/L))
API	Age (years): ≤30 = 0, 31-40 = 1, 41-50 = 2, 51-60 = 3, 61-70 = 4, >70 = 5 PLT count ( × 10^9^/L): ≥225 = 0, 200-224 = 1, 175-199 = 2, 150-174 = 3, 125-149 = 4, <125 = 5 API is the sum of age and platelet count (possible value 0-10)

*AAR – AST to ALT ratio; ALB – albumin; ALT – alanine aminotransferase; API – age-PLT index; APRI – AST to PLT ratio index; AST – aspartate transaminase; FIB-4 – age-AST-PLT-ALT index; Fibro-Q – age-AST-INR-PLT-ALT index; GGT – γ-glutamyl transpeptidase; GPRI – GGT to PLT ratio index; INR – international normalized ratio; PLT – platelet; S index – GGT-PLT-ALB index; ULN – upper limit of normal.

### Statistical analysis

Baseline patients’ characteristics are presented as mean ± standard deviation or median (interquartile range), and categorical variables as number (percentage). Statistical analyses were carried out using SPSS v. 18.0 software (SPSS Inc., Chicago, IL, USA) and STATA statistical package (release 11, 1, 2010, Stata Corporation, College Station, TX, USA). Normality of distribution was assessed using the Kolmogorov-Smirnov test. Univariate analysis (*t*-test or Mann-Whitney test as appropriate) was carried out to identify variables that differed significantly between patients with and without significant fibrosis. Correlations were calculated using Spearman test. Combined models were obtained by logistic regression. A value of *P* < 0.05 was considered statistically significant. The performance of the serological models was evaluated by their specificity and sensitivity as well as the area under receiver operating characteristic (ROC) curves. Cut-off values were determined according to the Youden index (21).

## Results

Forty-six patients were excluded from the study for the following reasons: 16 patients had invalid LSM values because of low success rates or high interquartile ranges, 5 had consumed >40 g/d of alcohol for at least 10 years, 5 had hepatitis C virus or hepatitis E virus coinfection, 8 had hepatocellular carcinoma, and 12 had exacerbations of hepatitis. The remaining 262 patients were enrolled (Table 2).

**Table 2:** Baseline characteristics of patients with chronic hepatitis B*^†^

Characteristics, median (IQR)	Total	F0-F1	F2-F4	Statistic	*P* value
Male (n, %)	198/262 (75.6)	126/171 (73.4)	72/91 (79.1)	*t* = 0.97	0.330
Age (year), mean ±SD	35.8 ± 10.9	35.8 ± 11.0	35.8 ± 10.6	*t* = 0.02	0.925
BMI (kg/m^2^), mean ±SD	22.9 ± 3.3	23.0 ± 3.8	22.6 ± 2.4	*t* = 5.9	0.766
PLT (10^9^/L)	114 (90-155)	125 (93-165)	97 (86-141)	Z = 3.2	0.001
AST (IU/L)	37 (27-49)	33 (26-44)	41 (32-56)	Z = 3.6	<0.001
ALT (IU/L)	48 (32-68)	44 (31-66)	57 (36-75)	Z = 2.8	0.009
GGT (IU/L)	29 (19-55)	25 (16-41)	49 (24-82)	Z = 5.3	<0.001
ALP (IU/L)	74 (62-95)	72 (61-90)	80 (64-103)	Z = 2.1	0.046
Globulin (g/L), mean ±SD	29.5 ± 4.4	29.4 ± 4.3	29.5 ± 4.7	*t* = 1.1	0.768
ALB (g/L), mean ±SD	44.3 ± 5.7	45.0 ± 4.8	42.9 ± 7.0	*t* = 10.4	0.008
TBIL (μmol/L)	14 (11-20)	13.8 (10.7-18.2)	15.4 (11.0-26.1)	Z = 1.8	0.099
INR	0.93 (0.88-1.06)	0.91 (0.86-1.00)	1.01 (0.91-1.11)	Z = 4.3	<0.001
LSM (kPa)	7.8 (5.6-11.7)	6.5 (4.9-8.9)	7.1 (7.1-16.9)	Z = 6.2	<0.001

*ALB – albumin; ALT – alanine aminotransferase; ALP – alkaline phosphatase; AST – aspartate aminotransferase; BMI – body mass index; GGT – γ-glutamyl transpeptidase; LSM – liver stiffness measurement; PLT – platelet count; TBIL – total bilirubin; SD – standard deviation; INR – international normalized ratio; IQR – interquartile range.

†Statistics and *P* values were calculated between patients with liver fibrosis F0-F1 and those with F2-F4.

The majority of the 262 eligible patients were male (198, 75.6%) and middle-aged (35.8 ± 10.9 years), with a mean body mass index (BMI) of 22.9. The distribution of each fibrosis stage was as follows: F0, 77 (29.38%) patients; F1, 94 (35.88%); F2, 51 (19.47%); F3, 19 (7.25%); and F4, 21 (8.02%). Therefore, 91 patients (34.73%) had significant liver fibrosis (F2-F4). Patients with non-significant fibrosis (F0-F1) and those with significant fibrosis (F2-F4) had no significant differences in baseline characteristics, including sex, age, BMI, globulin, and TBIL (all *P* > 0.05); but they had significant differences in other assessed variables, including ALT, AST, ALB, GGT, PLT, ALP, INR, and LSM (all *P* < 0.05).

### Correlations of serum models with histological finding and LSM values

S index and GPRI had the highest correlations (r = 0.373, *P* < 0.001; r = 0.372, *P* < 0.001, respectively), FIB-4, APRI, and API had moderate correlations (r = 0.199, *P* = 0.001; r = 0.197, *P* = 0.001; r = 0.134, *P* = 0.030; respectively), and Fibro-Q and AAR had no correlation with histological finding of fibrosis stage (r = 0.133, *P* = 0.066; r = 0.01, *P* = 0.886; respectively). Correlation coefficients of S index, GPRI, FIB-4, APRI, API, Fibro-Q, and AAR with LSM values were 0.516 (*P* < 0.001), 0.513 (*P* < 0.001), 0.195 (*P* = 0.005), 0.167 (*P* = 0.015), 0.009 (*P* = 0.897), and −0.011 (*P* = 0.874), respectively.

### Performance of serum models in diagnosing significant liver fibrosis

**Figure 1:**
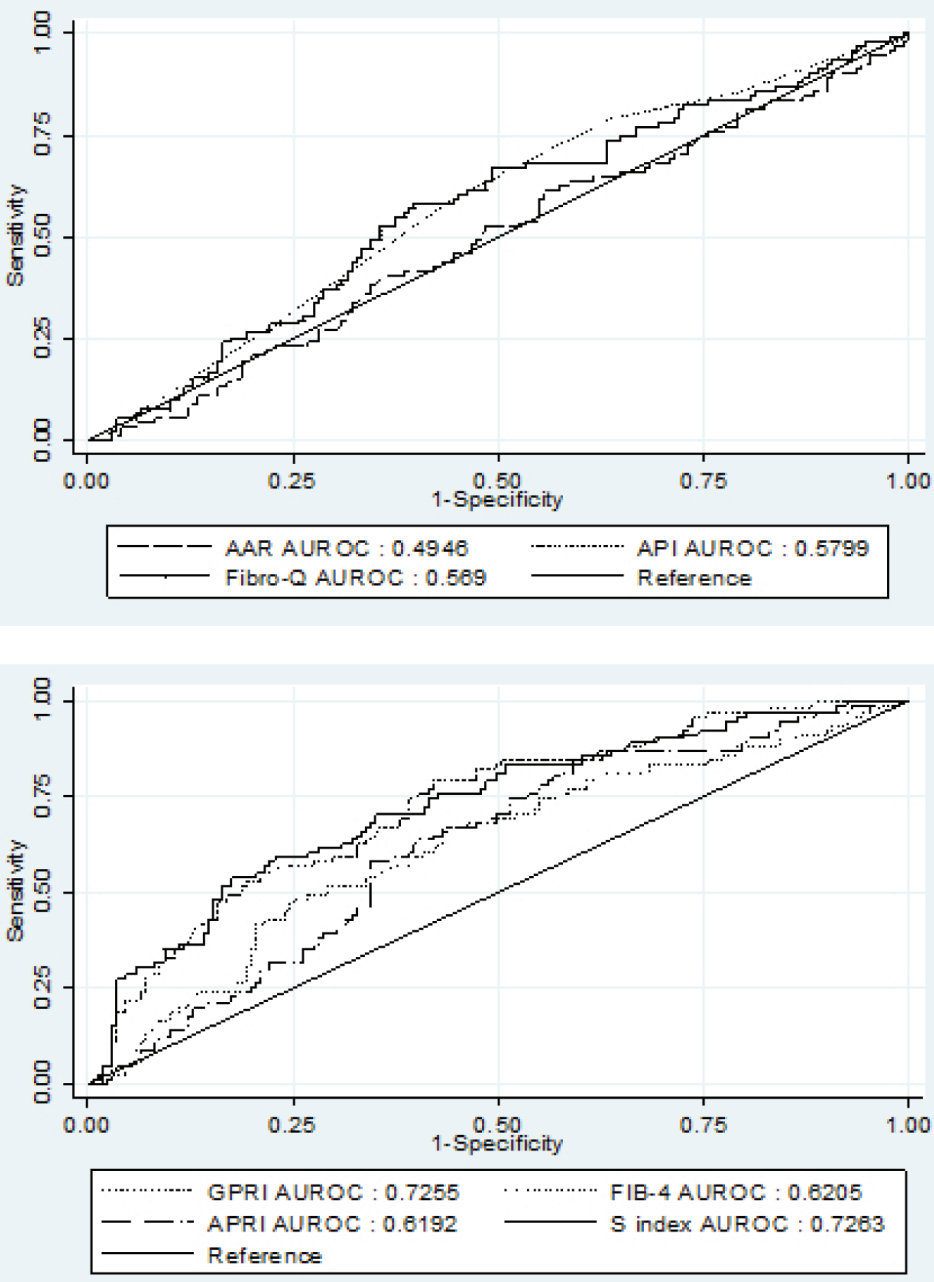
Receiver operating characteristic curves of serum models for predicting significant liver fibrosis. Serum liver fibrosis models, including age-platelet (PLT) index (API), aspartate transaminase (AST) to alanine aminotransferase (ALT) ratio (AAR), AST to PLT ratio index (APRI), γ-glutamyl transpeptidase (GGT) to PLT ratio index (GPRI), GGT-PLT-albumin index (S index), age-AST-PLT-ALT index (FIB-4), and age-AST-PLT-ALT-international normalized ratio index (Fibro-Q) showed different results in diagnosing significant liver fibrosis in patients with chronic hepatitis B. Judging from the area under the receiver operating characteristic curves, S index and GPRI had the best diagnostic value, followed by FIB-4, APRI, API, Fibro-Q, and AAR.

AUROCs of S index, GPRI, FIB-4, APRI, API, Fibro-Q, and AAR for predicting significant liver fibrosis were 0.726, 0.726, 0.621, 0.619, 0.580, 0.569 and 0.495, respectively (Figure 1 and Table 3). Univariate logistic regression model showed that LSM values (odds ratio [OR] 1.173, 95% CI 1.099-1.252, *P* < 0.001), GPRI (OR 1.551, 95% CI 1.045-2.303, *P* = 0.029), and S index (OR 1.603, 95% CI 0.960-2.677, *P* = 0.071) best predicted significant liver fibrosis in patients with CHB (Table 4). Because of their good diagnostic performance, LSM values, S index, and GPRI were subjected to further analysis. According to the maximum Youden index, optimal cut-off values of LSM, S index, and GPRI for predicting significant liver fibrosis were 10.7, 0.1369, and 0.2181, respectively (Table 5).

**Table 3:** Diagnostic performance of liver fibrosis models*

Models	AUROC	Standard error	*P* value	95% confidence interval	
				Lower bound	Upper bound
LSM	0.757	0.034	<0.001	0.690	0.823
S index	0.726	0.033	<0.001	0.662	0.791
GPRI	0.726	0.032	<0.001	0.662	0.789
FIB-4	0.621	0.037	0.001	0.549	0.692
APRI	0.619	0.035	0.001	0.550	0.688
API	0.580	0.037	0.033	0.508	0.652
Fibro-Q	0.569	0.037	0.066	0.496	0.642
AAR	0.495	0.037	0.886	0.421	0.568

*AAR – aspartate transaminase (AST) to alanine aminotransferase (ALT) ratio; API – age-platelet (PLT) index; APRI – AST to PLT ratio index; AUROC – area under the receiver operating characteristic curve; FIB-4 – age-AST-PLT-ALT index; Fibro-Q – age-AST- international normalized ratio (INR)-PLT-ALT index; LSM – liver stiffness measurement; GPRI – γ-glutamyl transpeptidase (GGT) to PLT ratio index; S index – GGT-PLT -albumin index.

**Table 4:** Odds ratios of fibrosis models determined by logistic regression analysis*

Models	Odds ratios (95% confidence interval)	*P* value
LSM	1.173 (1.099-1.252)	<0.001
S index	1.603 (0.960-2.677)	0.071
GPRI	1.551 (1.045-2.303)	0.029
FIB-4	1.054 (0.937-1.186)	0.379
APRI	1.002 (0.935-1.074)	0.954
API	1.145 (0.994-1.145)	0.060
Fibro-Q	0.999 (0.995-1.003)	0.662
AAR	0.750 (0.463-1.214)	0.242

**Table 5:** Optimal cut-off values of models in diagnosing significant liver fibrosis*

Models	Cut-off values	Specificity (%)	Sensitivity (%)	Youden index^†^	NLR	PLR	NPV (%)	PPV (%)
LSM	10.7	85.5	53.2	0.387	0.548	3.666	75.2	68.9
S index	0.1841	77.65	59.78	0.374	0.518	2.675	77.65	57.61
GPRI	0.2343	58.24	79.35	0.376	0.355	1.899	83.19	48.95

*APRI – aspartate transaminase (AST) to platelet (PLT) ratio index; FIB-4 – age-AST-PLT-alanine aminotransferase (ALT) index; GPRI – γ-glutamyl transpeptidase (GGT) to PLT ratio index; NLR – negative likelihood ratio; NPV – negative predictive value; PLR – positive likelihood ratio; PPV – positive predictive value; S index – GGT -PLT-albumin index.

†max (sensitivity + specificity −1).

### Performance of combined models in diagnosing significant fibrosis

**Figure 2:**
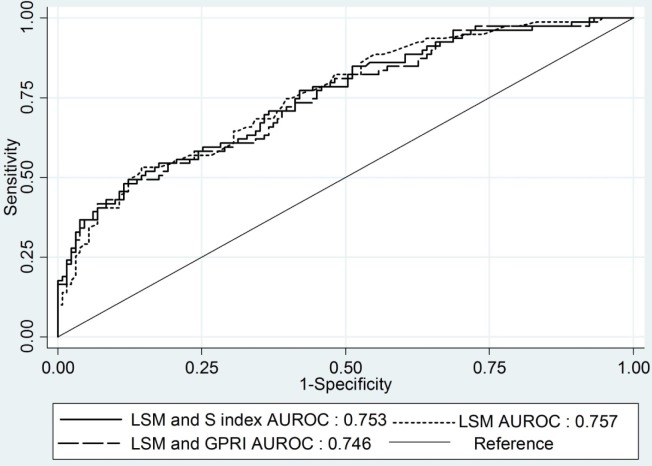
Receiver operating characteristic curves for combined models in predicting significant liver fibrosis. Liver stiffness measurement (LSM), and combined models of LSM plus either γ-glutamyl transpeptidase (GGT)- platelet (PLT)- albumin index (S index) or GGT to PLT ratio index (GPRI) showed good predictive value for significant liver fibrosis.

When the three most valuable models (LSM with S index or GPRI) were combined, the AUROCs for predicting significant liver fibrosis were 0.753 (*P* < 0.001) and 0.746 (*P* < 0.001), with sensitivities of 78.5% and 77.2%, respectively. A combination of either of these two models with LSM (S index + LSM, GPRI + LSM) improved the performance to a better level than that achieved by the S index or GPRI alone (AUROCs: 0.753 vs 0.726, 0.746 vs 0.726, respectively) (Figure 2). However, the diagnostic performance of these combinations was not significantly better than that of LSM (AUROCs: 0.753 vs 0.757, 0.746 vs 0.757, respectively).

## Discussion

Our study showed that S index and GPRI had the best diagnostic accuracy performance for significant liver fibrosis and were robust serum prediction models of significant liver fibrosis in patients with CHB. Liver fibrosis is considered a regenerative response to liver injury caused by increased production and decreased destruction of the extracellular matrix. In patients with CHB, a pathological finding of significant liver fibrosis indicates the need for immediate treatment. The major models of evaluating liver fibrosis in patients with CHB are liver biopsy, FibroScan, and serum biomarkers, the latter being more frequently available in primary care settings than the other two.

Dozens of serum liver fibrosis models have been developed and validated in clinical practice, all of them noninvasive, low-cost, and with AUROCs 0.50 ~ 0.86 (10). However, some serum models include biomarkers that are not routinely available, such as haptoglobin in Fibrotest (22) and α2-macroglobulin in Fibroscore (23), which is why many hospitals do not perform them. Furthermore, these models entail greater financial cost. In this study, we selected seven fibrosis models that include only routine laboratory test and are easily calculated. Also, most of them have frequently been used to assess liver fibrosis in patients with chronic hepatitis C (CHC). Because CHC and CHB differ greatly in terms of the histological changes in the liver and mechanisms that trigger fibrosis (6), models used in patients with CHC should be validated in CHB patients. We compared the predictive value of these seven models in the same sample of patients with the same judgment standard.

The highest performance for differentiating significant fibrosis in patients with CHB was found for LSM, followed by S index, GPRI, APRI, and FIB-4. On the other hand, API, Fibro-Q, and AAR showed poor or no predictive value. Logistic regression analysis showed that LSM values and GPRI were predictors of significant liver fibrosis. It should be noted that the S index (*P* value of odds ratio 0.071) was a borderline significant factor, meaning that it can be a good marker of significant fibrosis if the sample size is large enough, just as was found in the study by Zhou et al (12). The models that correlated best with histological scores were LSM, S index, and GPRI. However, Fibro-Q (*P* = 0.066) could also have significantly correlated with fibrosis stage if the sample size had been larger or distribution of patients in different fibrosis stages more even. Further analysis revealed that LSM values correlated more strongly with S index and GPRI than with other models. Finally, our findings indicated that S index and GPRI were the best models for diagnosing significant liver fibrosis. Castera et al (24) demonstrated that combinations of LSM and other serum fibrosis models could avoid the need for liver biopsy in more than two thirds of patients with CHB. In our study, combinations of S index plus LSM or GPRI plus LSM better predicted significant fibrosis than either S index or GPRI alone. One explanation is that combinations of LSM (a model with a low false-positive result) with GPRI or S index (models with high false-negative results) were able to reduce the incidence of both false negative and false-positive results, thus improving diagnostic performance. Diagnostic sensitivity of the combined models was more than 20% better than LSM alone, even though the AUROCs did not improve.

The S index includes GGT, PLT, and ALB, whereas GPRI only includes GGT and PLT. These scores can differentiate liver fibrosis and can be simply calculated by straightforward formulas. Serum ALB and GGT concentrations and PLT count differ significantly between patients with F0-F1 and those with F2-F4 liver fibrosis. With progression of fibrosis, the decreased ability of hepatocytes to synthesize ALB leads to a decrease in serum ALB concentrations, which is why serum ALB can serve as an indirect indicator of liver fibrosis. Serum GGT, on the other hand, can be an independent predictive marker of liver fibrosis, since it is not affected by changes in ALT or TBIL (25). The splenic platelet pool may be greatly increased in the presence of splenomegaly, since up to 50%-90% of platelets are sequestered in the spleen. This redistribution of cells from the peripheral circulation to the spleen can result in thrombocytopenia (26). On the other hand, as liver disease progresses from inflammation to fibrosis and finally to cirrhosis, decreased production of thrombopoietin associated with hepatocellular damage may contribute to exacerbation of thrombocytopenia (27). In addition, antibody-mediated platelet destruction (28) and myelotoxic effects (29) can also cause decreased platelet counts.

APRI, FIB-4, AAR, and Fibro-Q contain several inflammatory biomarkers, including AST or ALT. Fluctuating patterns of AST or ALT may limit the applicability of these models in patients with CHB, which is not the case in patients with CHC (30,31). API was first implemented in patients with CHC and one of its two variables is age, which in our study did not correlate with liver fibrosis stage. This may explain why in our study AUROC for API was lower than in other reports (32,33).

In conclusion, we found that S index and GPRI were the simple and most useful of the seven models for prediction of significant liver fibrosis in patients with CHB, which is of particular importance in the settings where FibroScan is unavailable. Combining LSM and either S index or GPRI seems a promising approach that may increase the performance for diagnosis of significant liver fibrosis.
